# An optoelectronic implantable neurostimulation platform allowing full MRI safety and optical sensing and communication

**DOI:** 10.1038/s41598-024-61330-w

**Published:** 2024-05-15

**Authors:** Pascal Doguet, Jérôme Garnier, Aurore Nieuwenhuys, Carmen Godfraind, Yohan Botquin, Antoine Lemaire, John Justice, Antoine Nonclercq, Riëm El Tahry, Brian Corbett, Jean Delbeke

**Affiliations:** 1 Irisia SRL, Court-Saint-Etienne, Belgium; 2 Synergia Medical, Mont-Saint-Guibert, Belgium; 3 UPVD (PROMES-CNRS), Perpignan, France; 4 Tyndall National Institute, University College, Cork, Ireland; 5 Bio-, Electro- and Mechanical Systems (BEAMS), Universite Libre de Bruxelles, Bruxelles, Belgium; 6 Department of Neurology, Institute of Neurosciences (IONS), Universite Catholique de Louvain, Cliniques Universitaires Saint Luc, Bruxelles, Belgium; 7 Academic Medical Consultant, Brussels, Belgium

**Keywords:** Neuroscience, Biomedical engineering

## Abstract

A novel programmable implantable neurostimulation platform based on photonic power transfer has been developed for various clinical applications with the main focus of being safe to use with MRI scanners. The wires usually conveying electrical current from the neurostimulator to the electrodes are replaced by optical fibers. Photovoltaic cells at the tip of the fibers convert laser light to biphasic electrical impulses together with feedback signals with 54% efficiency. Furthermore, a biocompatible, implantable and ultra-flexible optical lead was developed including custom optical fibers. The neurostimulator platform incorporates advanced signal processing and optical physiological sensing capabilities thanks to a hermetically sealed transparent nonmetallic casing. Skin transparency also allowed the development of a high-speed optical transcutaneous communication channel. This implantable neurostimulation platform was first adapted to a vagus nerve stimulator for the treatment of epilepsy. This neurostimulator has been designed to fulfill the requirements of a class III long-term implantable medical device. It has been proven compliant with standard ISO/TS10974 for 1.5 T and 3 T MRI scanners. The device poses no related threat and patients can safely undergo MRI without specific or additional precautions. Especially, the RF induced heating near the implant remains below 2 °C whatever the MRI settings used. The main features of this unique advanced neurostimulator and its architecture are presented. Its functional performance is evaluated, and results are described with a focus on optoelectronics aspects and MRI safety.

## Introduction

This research falls within the scope of neurostimulation, one of the fastest-growing areas of medicine, that has been approved for the treatment of several debilitating conditions, including epilepsy, chronic pain, Parkinson’s disease, major treatment-resistant depression, gastroparesis and essential tremors, and that is currently undergoing investigation for use in other indications, such as obesity, migraine and heart failure^1,2^. Neurostimulators are small implantable devices that generate electrical impulses on nerves or neural tissue, sent through electrode wires to replace, modulate or modify central and peripheral nervous system functions. Implantable neurostimulation devices are increasingly being used to treat several chronic disorders and restore impaired functions and the field of applications is broadening. In the past decade, neurostimulation has witnessed significant advances with regards to clinical applications and technology development, coupled with the rapid growth of the neurostimulation industry, improvements in current devices, and the development of next generation neurostimulation systems. The concept of neurostimulation as a therapeutic option for patients is not new, but has growing interest, owing to the limitations of pharmacological intervention and the therapeutic advantages that such devices can offer.

Despite these advances, one major problem remains regarding the safety related to undergoing a magnetic resonance imaging (MRI) examination with a neurostimulator. MRI diagnosis is currently forbidden or bound by very strict rules due to the presence of electrically conductive materials and/or materials exhibiting magnetic properties in a neurostimulator, mainly its metallic casing and wires between the neurostimulator casing and the electrodes. During MRI, this can lead to unwanted stimulation, pain and burns^3–6^ and so deprive some patients from access to a major diagnostic and monitoring instrument. Also, the use of MRI is often a key tool for researchers that aim for therapy improvement and the need to see, in real-time, the impact of stimulation on brain functions.

## Background and related work

A classical design scheme for an implantable neurostimulator is composed of the neurostimulator itself, namely the implantable pulse generator (IPG), with electronics and battery enclosed in a titanium casing, connected to a lead incorporating wires delivering stimulation current to the electrodes at the stimulation site. When undergoing MRI with an implantable neurostimulator, there are several risks and considerations to be aware of^7^:

- Heating or Burns: During an MRI, the radiofrequency (RF) energy used to generate images can cause overheating of conductive elements. It can potentially result in burns or tissue damage, especially with long lead wires acting as an RF field antenna. This overheating is the primary concern while performing MRI with an implantable neurostimulator.
- Device Movement or Displacement: The strong magnetic field can cause movement or displacement of the implantable neurostimulator, leading to pain or discomfort for the patient.
- Magnetic Field Interference: MRI machines generate strong magnetic fields that can potentially interfere with the device's functioning. This interference can result in the device malfunctioning or delivering unintended stimulation.
- Programming or Function Changes: The electromagnetic fields generated during an MRI can potentially alter the programming or settings of the neurostimulator device. This may affect the therapeutic benefits provided by the device or lead to unintended changes in stimulation parameters.
- Image Artifact: The presence of the implantable neurostimulator can create artifacts on the MRI images, making it challenging to interpret the scans accurately. These artifacts can obscure the visualization of the surrounding tissues and structures. This is called MRI compatibility.

To minimize these risks, it is crucial to inform the MRI technician and radiologist about the presence of an implantable neurostimulator before undergoing the procedure. Either MRI is just forbidden and, sometimes, patients need to be explanted and reimplanted to undergo MRI, or the device is marked as MRI conditional: appropriate precautions and necessary adjustments are needed to ensure the patient’s safety: such as reduced MRI power, change of coils, forbidden areas to scan in the vicinity of electrodes (such as the brain), etc. Even with these precautions, these limitations will most often affect the obtainable image quality or simply make it impossible to visualize the area of greatest interest, such as the brain, if electrodes or lead are nearby. So, although MRI has become one of medicine’s most important diagnostic tools, the safety concerns can make MRI very tedious, contraindicated or forbidden by both device and MRI equipment manufacturers for patients with an implantable neurostimulation device, and more generally with an active implanted medical device (AIMD).

Manufacturers of implantable neurostimulators used several design approaches to reduce the risks posed by MRI^8^, such as the use of non-magnetic materials in the construction of the neurostimulator to prevent magnetic field interactions, reduction of the length of the lead or the size of the neurostimulator when it is possible and adding bandstop filters in the lead conductive path presenting a high impedance at the MRI pulsed RF frequency. Still, the presence of conductive wires in the lead remains a safety threat.

Here we present a novel approach for the design and realization of an implantable neurostimulator based on optics and optoelectronics that, to the best of our knowledge^3–7^, has never been proposed before. The wires usually used to convey electrical current from the neurostimulator to the electrodes are replaced by optical fibers. Photovoltaic cells at the tip of the fibers convert monochromatic optical energy to electrical impulses. Furthermore, a biocompatible, implantable and ultra-flexible optical lead was developed with a custom optical fiber. Also, the neurostimulator platform incorporates advanced signal processing and optical physiological sensing capabilities thanks to developing a hermetically sealed transparent nonmetallic casing. Transparency also allowed the development of a high-speed optical communication crossing the skin barrier. Eventually, in order to meet the requirements of increasing therapy sophistication such as closed-loop methods or continuous sensing, more power can be made available, and autonomy increased with the recharging scheme that was developed.

This implantable neurostimulation platform, called NAO, provides a clinically viable solution that can be tuned to any neurostimulation application. It was first adapted to the treatment of epilepsy through vagus nerve stimulation (VNS), which is described in this study.

## Material and methods

**Figure 1:**
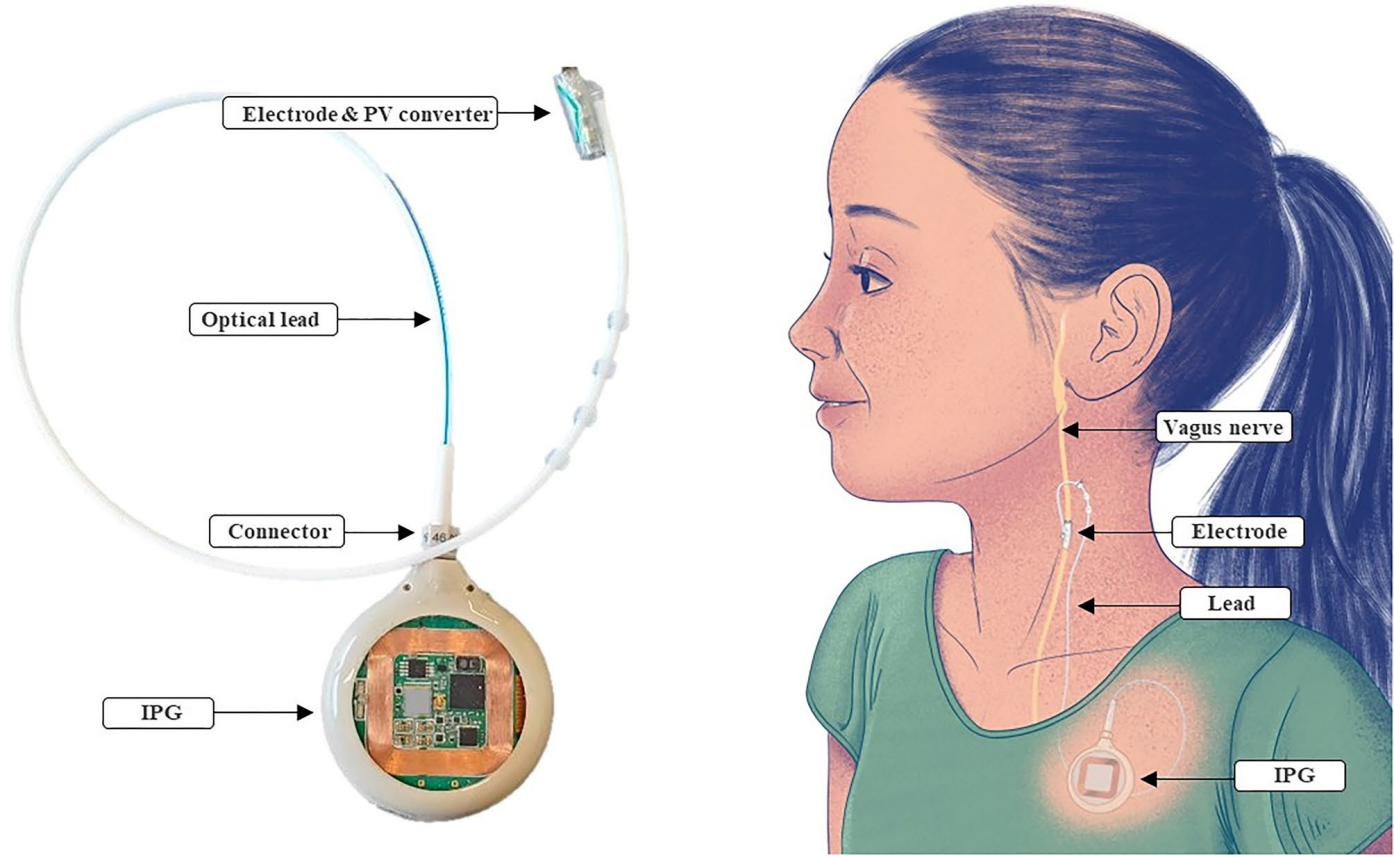
Implantable neurostimulator for Vagus Nerve Stimulation and its main components, showing the implantation site.

The NAO∙VNS implantable neurostimulator (Fig. 1) was developed to stimulate the vagus nerve for long-term human implantation. It consists of an IPG with a 40 mm diameter silicone-coated fused silica casing with transparent surfaces. The casing is fitted on the side with a female optical connector partly made from a biocompatible polymer and fused silica. The optical lead consists of a connector to the casing, and, at the distal end, it presents a photovoltaic converter and a self-sizing neural cuff electrode. The neurostimulator provides a single stimulation channel of up to 3 mA, is powered by a 24 mAh rechargeable battery and can be recharged through an inductive link providing up to 200 mA of recharging current using an external charger. Data transmission is ensured by optical means between the implantable neurostimulator and its external charger. The charger also acts as a data relay to an external programming device. The communication between the charger and the programming device relies on wireless Bluetooth communication. The design of the NAO∙VNS system considers all the constraints of a class III long-term implantable medical device, following the European Medical Device Regulation (MDR) and FDA regulations^9,10^. In this paper, we will mainly focus on the implantable parts of the system and, specifically, the optoelectronic chain for stimulation and its positive impact on MRI safety and compatibility.

### Optoelectronic chain for stimulation

#### General overview

**Figure 2:**
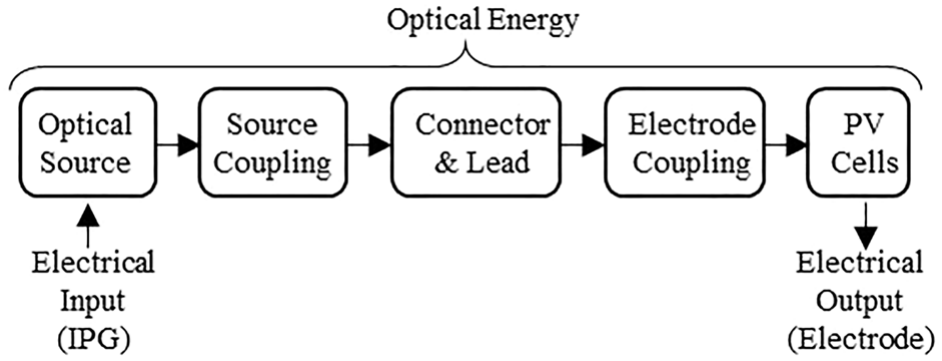
Optoelectronic chain and its constituting elements; input and output energies are electrical; energy transport from IPG to electrode is optical through optical connector and fibers.

The optoelectronic chain represents an innovative way to transport stimulation signals energies from the IPG towards the electrodes. It relies on electrical to optical energy conversion at the implant site, and vice versa, at the electrode location, as illustrated in Fig. 2.

This optoelectronic chain is made up of different components. An optical source, integrated into the implanted IPG, converts input electrical energy into light. A source coupling element ensures the light transmission from the optical source towards the optical fiber cable. It ensures energy transfer and constitutes a mechanical binding element between the optical source and the lead, as further discussed in section IIIA3. The optical lead transports physical energy using optical fibers. Its proximal end is made of an optical connector, allowing connection and disconnection between the IPG and the lead. Disconnection is necessary for surgery and potential replacement. At the distal end of the lead, an electrode coupling element links the tip of the optical fiber to the optical power converter. Laser power conversion for current delivery through the electrode contacts is achieved with a photovoltaic cell adapted to the laser spectrum in order to optimize efficiency.

The developed concept solves the MRI issues encountered in MRI due to conductive wires by replacing them with optical fibers, while still providing programmable stimulation impulses at the electrodes. Typical VNS use a current generator circuit with output range reaching up to 3 mA with a resolution equal to or above 8 bits and a voltage compliance allowing the IPG to deliver the selected current on an impedance load of a few kiloOhms^11^. It usually requires a high-voltage custom integrated circuit reaching around 10 V. Also, removing charges accumulated by the stimulation pulse at the electrode-tissue interface is necessary to prevent electrochemical damage. Therefore, stimulation pulses are made biphasic with a stimulation phase that is always accompanied by a phase of opposite polarity whereby injected charges are recovered. The recovery can be either passive (shortening the electrodes via a resistance) or active (current of oppositive polarity) or made of a combination of both^12,13^.

**Figure 3:**
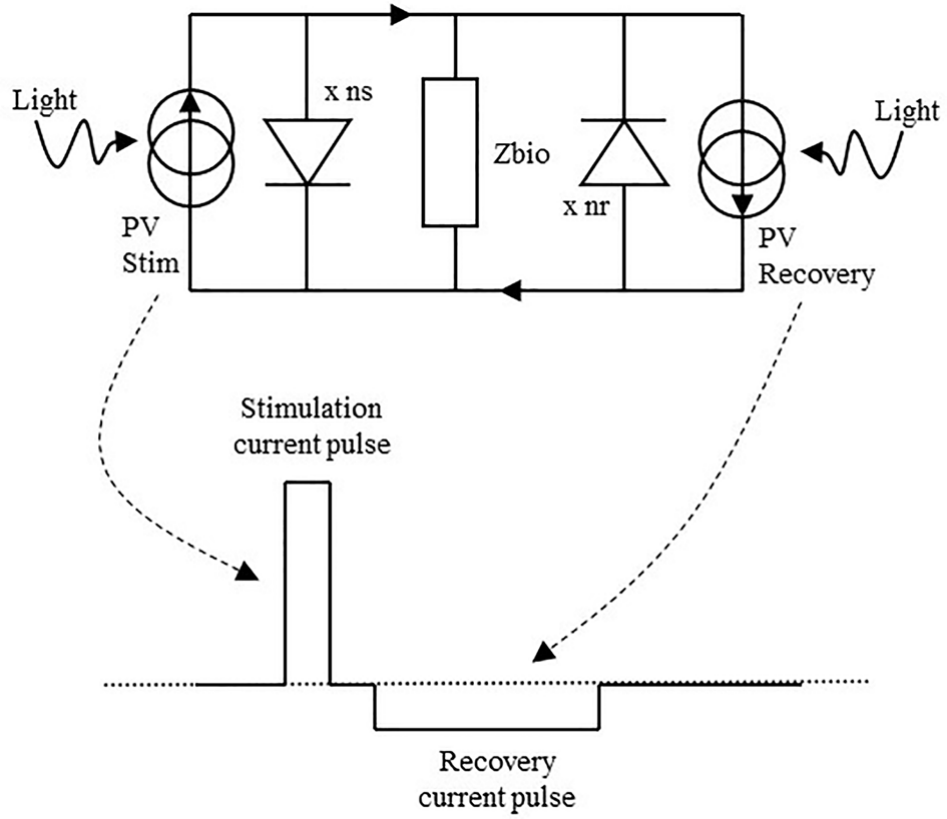
Equivalent electrical scheme of the photovoltaic conversion. Each PV cell is in essence a current source with a voltage compliance governed by the number of diodes in series. Lighting up the different PV cells separately allows generating the stimulation and recovery currents needed.

The chosen approach is directly based on the properties of a semiconductor photovoltaic (PV) cell. Electrically, a PV cell is equivalent to a current generator in parallel with a diode (Fig. 3). Increasing the intensity of the light increases the photocurrent, which varies linearly up to high irradiation levels and beam concentration factors. Thus, the cell can be exploited as a controlled current source by varying in a controlled manner the intensity of the light-emitting source. A single PV cell's open circuit voltage *Voc* is typically 0.5 V for silicon and around 1 V for Gallium Arsenide. This is not a sufficient voltage compliance to inject a stimulation current of a few mA in an expected load impedance of a few kiloOhms (e.g., 1 mA × 2 kΩ = 2 V). By integrating PV cells with similar characteristics in series (x ns and x nr in Fig. 3), the generated current remains similar, but the voltage compliance is increased by a factor equivalent to the number of cells. Next, to allow for a recovery phase, two similar photovoltaic cells are added in a back-to-back connection. With *Zbio* representing the output impedance (biological tissue to stimulate and electrode contact interface), the complete equivalent electrical scheme of the photovoltaic conversion can be drawn as Fig. 3.

**Figure 4:**
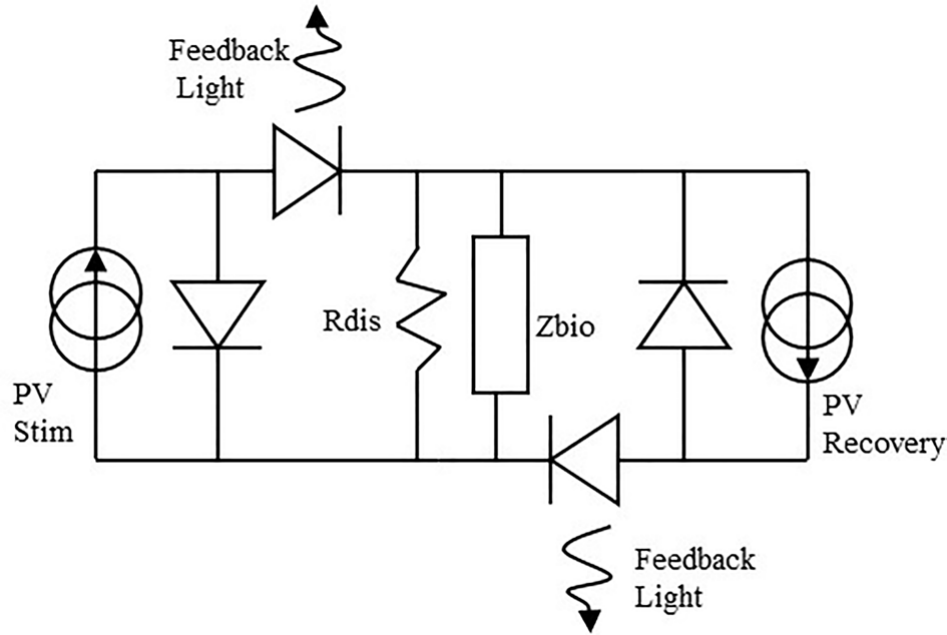
Circuitry at the electrode, adding a discharge resistor for residual charges and two feedback LEDs in the current paths are added to the initial scheme.

Two optical sources send light pulses controlled in intensity and time at the IPG side, each with its own optical fiber. On the other side of the fiber, the two optical sources are each being coupled to separate photovoltaic cells generating bipolar current pulses at the electrode contacts. Figure 4 shows the circuit at the electrode, including both PV cells. This circuit is further enhanced by adding a discrete resistor *Rdis* of typically a few hundred kiloOhms allowing a passive discharge path of the injected charges that might remain after active recovery.

An opto-isolated monitoring of the current was also added to the photovoltaic circuitry (Fig. 4). Indeed, providing a means to check that current is delivered by the electrodes to the nerve is crucial for validating the success of the surgery as well as the short and long-term technical performance of the implant. The monitoring works in the opposite manner compared to stimulation. Here, a light emitting diode (LED) is placed in series with the PV cell at the electrode site. The current generated by the PV cell goes through the biological tissue and drives the LED for feedback measurement. The light emitted by the LED travels back to the IPG through a third fiber to illuminate a photodiode and provide a measure of the current flowing through the nerve.

#### Optical sources

**Figure 5:**
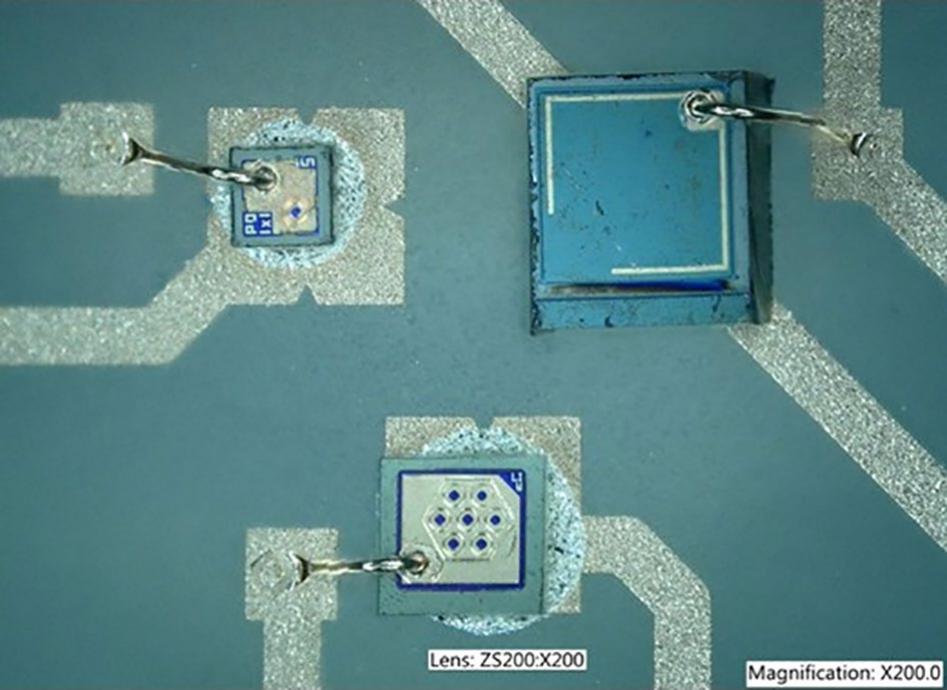
Photograph of the two VCSELs and photodiode assembled on the ceramic PCB in a triangular arrangement.

Vertical Cavity Surface Emitting Lasers (VCSELs) were chosen as the optical sources^14^. VCSELs possess performance advantages over both conventional LEDs and edge-emitting lasers: low threshold currents for high efficiency and low power consumption as necessary for a battery-based device; surface-normal emission with low quasi-circular divergence to efficiently couple light into the fiber; wavelength stability with temperature is an order of magnitude better. Other factors such as price, availability, reliability and size must also be considered. The VCSEL was found to offer the best balance between all those criteria. Custom VCSELs at a wavelength of 850 nm were manufactured with power conversion efficiency of up to 35% and a die size of less than 200 µm square. One device produces up to 25 mW of optical power through a total of 7 separate apertures and is dedicated to the stimulation phase. Another single aperture device deals with the recovery phase, typically 4 to 10 time lower in current but with a longer pulse duration, with a maximum generated optical power of 10 mW. Figure 5 shows the VCSELs mounted as bare dies on a custom ceramic PCB, together with the feedback photodiode.

#### Source-fiber optical coupling

**Figure 6:**
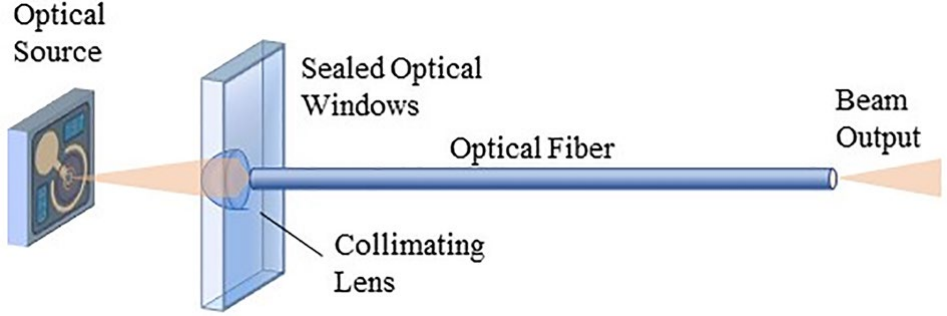
Schematic of the source-fiber coupling, not showing supporting elements such as ceramic PCB and connector.

Source-fiber coupling is the process by which light emitted by the optical sources is injected into the optical fiber. In this application, the optical fiber carries optical power, rather than, as usual in telecommunications, data. It needs to be performed as efficiently as possible, with minimal loss. As the beam emitted by the VCSELs is divergent (expanding), it is necessary to add some micro-optic elements, here lenses, to couple light into the fiber^15^ (Fig. 6).

**Figure 7:**
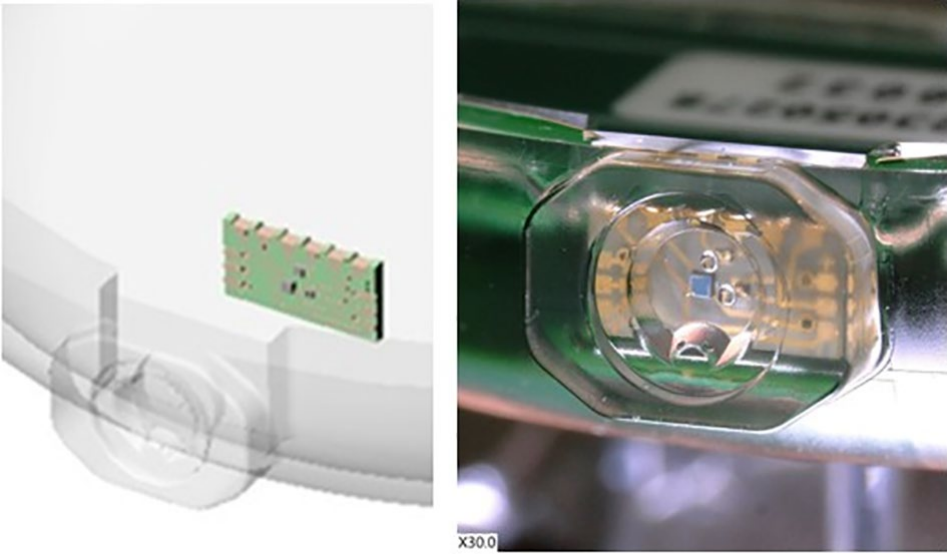
Micro-optic coupling (left) showing lenses and ceramic PCB recess; the ceramic PCB is placed within the header recess either with passive or active alignment and fixed with glue. The photograph (right) also shows the two lenses for each VCSEL.

Optical simulation software (OpticalRayTracer^16^ and Matlab^©^) were used to design the solution for this micro-optic coupling. The focus was on optimization of the lenses and making the design as robust as possible with regards to the different possible misalignments of the optical chain (VCSEL/lens misalignment, lens/fiber misalignment, PV cell/fiber misalignment) that can drastically affect the power transfer efficiency. Hyperbolic lenses were selected as they have the great advantage of suppressing the spherical aberration meaning that light radiating from a single point source can be totally focused into the fiber, when passing through such lens^17,18^. Lenses parameters, i.e. maximum lens radius and lens hyperbolic curvature factor, were optimized with the software. The lenses are embedded into the wall of the IPG encapsulation. Two lenses are needed, one for each VCSEL. These are manufactured with micrometer accuracy as a monolithic block of fused silica using subtractive 3D printing, together with a specific recess for the ceramic PCB (Fig. 7).

The recess is used to place the ceramic PCB as accurately as possible: VCSELs are aligned within less than 10 µm error with their respective lenses. Micro-optics coupling and ceramic PCB are also manufactured with micrometer accuracy and the VCSELs also need to be placed on the PCB with similar accuracy. The tolerances on the placement of the VCSELs and design of the micro-optics were designed to provide to the connector insert a sufficiently large ± 50 µm insertion tolerance.

#### Optical connector

The implantation of a neurostimulator is generally operated at two different surgical sites to limit its invasiveness. In the case of VNS, the implant encapsulation, because of its bulkiness, cannot be placed near the stimulation site in the neck^19^. That is why it is placed further away, at the subclavian level. The electrode is placed around the target nerve. The surgeon then creates a subcutaneous tunnel through which (s)he inserts the electrode lead until it reaches the implant encapsulation site. It is thus necessary to design a connecting element between the lead extremity and the implant encapsulation. Also, if the device must be explanted, for example because of battery exhaustion, the surgeon can unplug the connector from the encapsulation and replace the latter, while the electrode and its optical lead remain in place.

This optical connector had to be specifically designed to meet the very stringent requirements for the fused silica tip to match the cavity very accurately in the micro-optics of the IPG. Strain relief is used to relax any interface stress between the connector and the lead. The screw and connector body are made of PEEK polymer material. The connector also comprises a seal to prevent the ingress of biological tissue and water into the connector female cavity. The tolerance between the connector male plug and mating female cavity structures are very tight, in the order of microns, in order to maximize optical power transmission.

#### Optical lead

The optical lead consists of a silicone tubing with three tunnels, one for each optical fiber. These fibers need to meet very stringent requirements which led us, after market review and tests on available products, to develop our own custom optical fiber. For coupling purpose, an optical fiber with large Numerical Aperture (NA) is required. Another important usability criterium is the minimum bending radius a fiber can sustain without losing light in the bent. The minimal bending radius must also be as small as possible (< 1 cm) as the path of the optical lead, once implanted, includes very tight bends.

Implantability also requires the fiber to be very flexible and resistant to mechanical breaking, sustaining repetitive body movements for years. Furthermore, it needs to be made of biocompatible materials. The fiber's optical properties also need to be stable in time and not affected by water ingress through the lead. There are two main possibilities for optical fiber core material: glass and polymer. The glass transmits the VCSEL wavelength of 850 nm with nearly no losses, while the polymer sustains absorption. However, glass is brittle with poor flexibility, limiting it to core diameters below 100 µm, low NA, and high bending radius. On the other hand, polymeric fibers and their mechanical properties offer advantages over glass-based ones, such as high flexibility and mechanical resistance, higher NA available than silica, and higher diameter to allow optimal optical coupling into the fiber.

On the market, Polymethyl methacrylate (PMMA) is one of the most popular material for polymer optical fibers. PMMA is mainly used for the core, it is easy to manufacture and can have various diameters in the range of 75–1000 µm. The major drawback of PMMA is that it strongly absorbs water, which changes its mechanical and optical properties and leads to high transmission losses^21,22^. Other alternatives were investigated including hydrogel fibers, but these are either biodegradable (not fit for long term implantation) or have a too high absorption factor^23–25^.

A careful review of available polymer materials^26^ leading to the selection of a combination of Cyclic Olefin Polymer (COP) and a cladding in tetrafluoroethylene hexafluoropropylene and vinylidene fluoride (THV) providing a high numerical aperture around 0.72. This custom fiber was manufactured by thermal drawing. Both materials are biocompatible and hydrophobic. THV has already been used as a coating for medical devices^27^, and therefore has a history of biocompatibility. Likewise, COP is also used for medical equipment and containers. COP can be supplied as an ultra-high purity optical grade polymer and is widely used in optical applications where refractive index stability over high heat and humidity is crucial^28^. It is also used in medical and laser devices due to its exceptional chemical resistance, low fluorescence and optical stability. THV has excellent permeation resistance and the ability to be bonded to elastomers and hydrocarbon-based plastics. A polyvinylidene difluoride (PVDF) layer is added around the cladding to ease polishing and insertion into the silicone lead and act as protection.

The fiber has a core diameter of 230 µm, a cladding of 10 µm thickness and an overclad/jacket made of PVDF with 60 µm thickness, the external diameter of the fiber is 300 µm + /- 10 µm.

This fiber has a numerical aperture of 0.72 when a minimal NA of 0.5 is required for optimal coupling between the optical source and the fiber, considering the VCSEL optical divergence and associated micro-optics. COP attenuation in the wavelength range from 550 to 875 nm is less than 3.2 ± 0.42 dB/m which is acceptable, knowing that the fiber is 40 cm long.

#### Photovoltaic conversion

The photovoltaic cells meet specific requirements to generate the stimulation currents needed with VCSEL wavelength at 850 nm. The voltage compliance must be at least 4 V for the stimulation side, less for the recovery side. The maximum current generation must be above 2 mA and ideally around 3 mA and the overall photovoltaic die, including the two PV cells and feedback LEDs shall have a footprint smaller than 1 mm × 1 mm.

The efficiency of a direct bandgap PV cell is higher for wavelengths close to the band gap value, due to reduced thermalized losses of the photo-generated carriers^29–31^. As 850 nm corresponds to an energy of 1.46 eV, gallium arsenide (GaAs) material, which has a band gap of 1.42 eV (870 nm), is the perfect candidate for this application.

Under laser illumination, a single GaAs PV cell produces around 1 V. To generate a voltage compliance of 4 V and more, PV cells have to be connected in series: the total voltage compliance is equal to the sum of the cells in series, and the surface of a single PV cell dictates the generated current. Two strategies are available to realize this voltage compliance: either a multi-segment interconnection where the total available surface is divided into separated cells (as quarter in a pie) and connected in series; or a multi-junction approach where cells are vertically connected via tunnel junctions. Both have pros and cons^32^ and the choice was made for a multi-segment approach, with an epitaxy less risky and less complex to implement.

**Figure 8:**
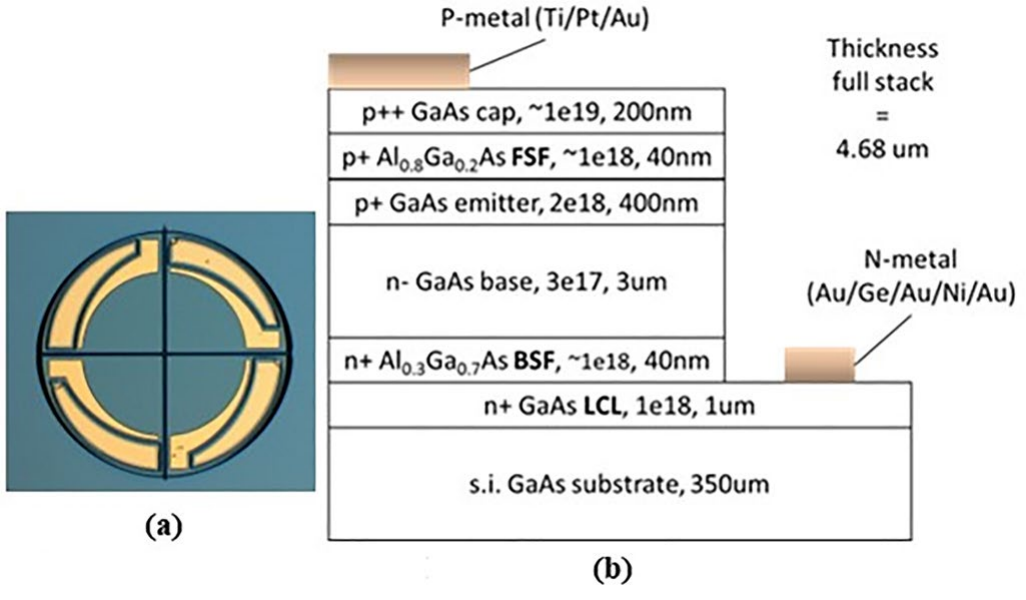
(**a**) Design of four interconnected PV cells and (**b**) epitaxy structure of the PV cell giving composition, doping level (cm^−3^) and thickness (nm) of each layer.

As the light from the optical fiber has a circular beam shape, the PV cell also needs to have a circular shape to maximize light collection. The simple design separates the circle into pie segments of equal size and interconnects them in series (Fig. 8a).

The photogenerated current is linearly dependent on the optical power illuminating the PV cell. With this fact, the optical power is simply adjusted to reach 3 mA. However, for sub-mm sized PV cells, 3 mA flowing through the structure could lead to series resistance effect. So, particular attention was paid to designing the the PV cells, especially interconnection paths and lengths. It is based on 50 mm diameter semi-insulating GaAs wafers. The epitaxial layer structure (see Fig. 8b) is a *p-n* homojunction with two AlGaAs front and back surface field layers (FSF, BSF) to limit electron and holes in the incorrect direction of electrons and holes respectively. In order to reduce the series resistance effect, a lateral conductive layer (LCL) is added on the substrate and metal fingers are added on top in order to shorten the conduction path for holes to be collected.

**Figure 9:**
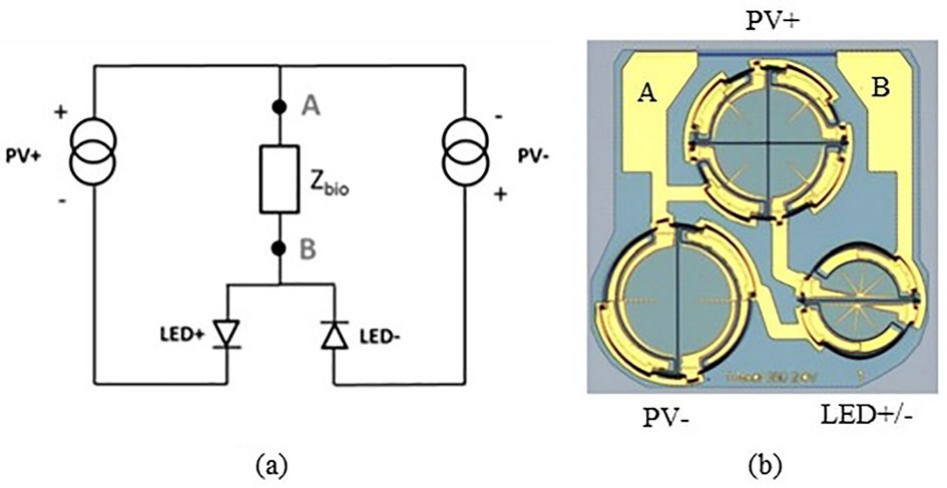
(**a**) Topographic electrical circuit of the photovoltaic die, including stimulation and recovery channels and feedback LEDs, Z_bio_ being the biological impedance; (**b**) Photovoltaic die, showing a stimulation channel consisting of four PV cells in series (at the top), while recovery channel has two (bottom left); the two LEDs are split each in half a circle (bottom right).

Figure 9 shows the resulting photovoltaic die (Fig. 9b) and its electrical equivalent circuit (Fig. 9a), including the two current generation channels, one for stimulation and one for recovery, two LEDs to provide some feedback and two large pads to allow connection to the electrode. Fitting all these devices on the footprint of 1.1 × 1.1 mm^2^ limits the PV cells diameter to around 400 µm.

#### Photovoltaic die coupling and encapsulation

The photovoltaic die encapsulation embeds the photovoltaic die just described. The encapsulation ensures optimal coupling by maintaining the fibers in tight alignment with the PV cells and LEDs of the photovoltaic and so minimizes power losses. The second function of the encapsulation is to protect the die from the environment and from mechanical stresses as well as providing hermeticity against body fluid ingress and protecting the body against non-biocompatible gallium arsenide diffusion.

**Figure 10:**
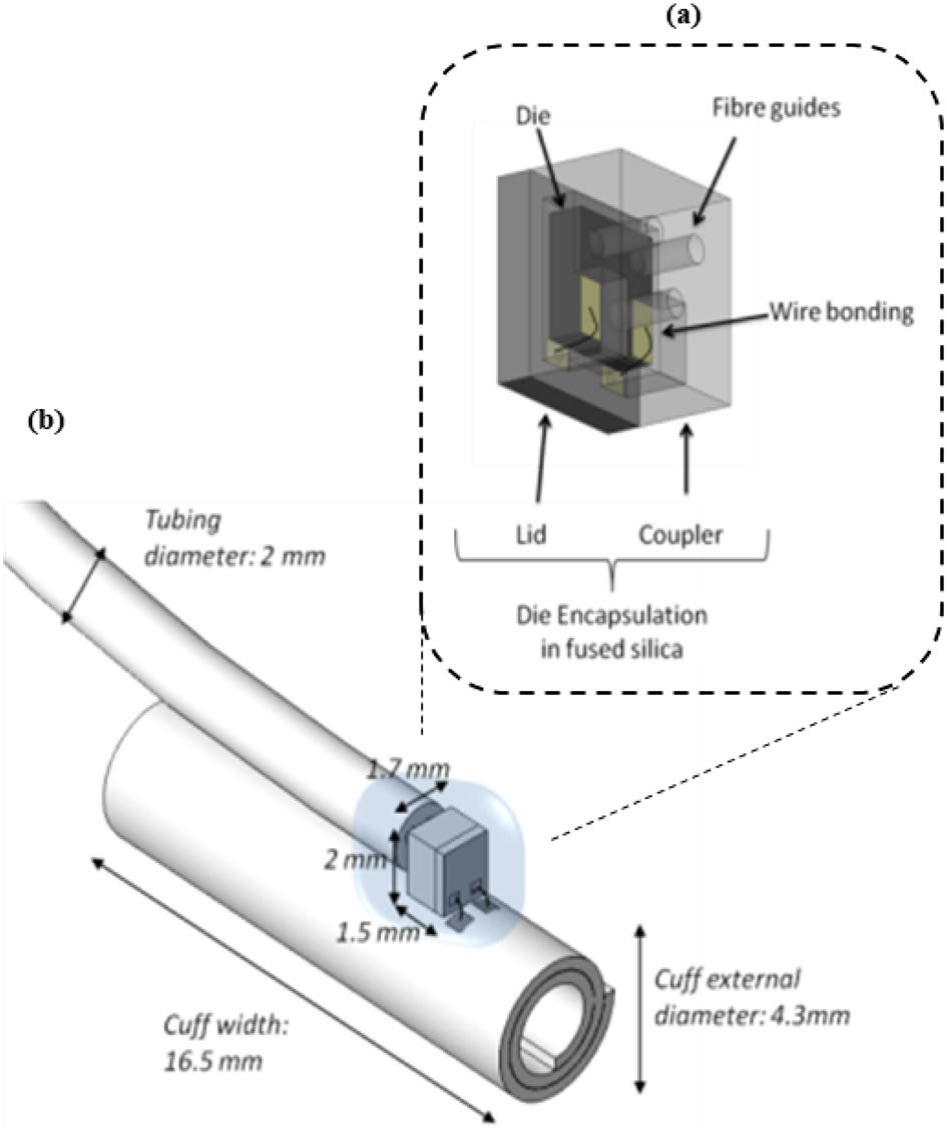
(**a**) Photovoltaic die encapsulation CAD model, consisting of two parts: lid and coupler; (**b**) Assembled Electrode, showing dimensions and placement of the encapsulation and connections.

The encapsulation is made of fused silica and is manufactured using laser patterning subtractive 3D printing. The encapsulation consists of two parts (Fig. 10a) that are hermetically laser welded together: a lid and a coupler. The coupler is so called as it integrates fiber guides for accurate (< 10 µm) alignment of the fibers with the PV cells. The fiber guide recess ends with a 35 µm hermetic window. Fibers are inserted within the guides, passively or actively aligned and glued with epoxy. The lid acts as a support for the PV die. The die is accurately placed on the lid and wire bonded to golden pads present on the lid. Specific gold vias formed through glass vias are embedded in the lid thickness. They provide hermetic electric connection between the inner pads next to the die and the outer pads on the other side of the lid.

The electrical connection between the outer pads and the contacts on the electrode is realized via gold wires and resistance welding (Fig. 10b). The encapsulation is further protected with a layer of silicone.

### Cuff electrode

The NAO∙VNS targets the stimulation of the vagus nerve in the neck area. Different kinds of electrodes are used to stimulate peripheral nerves, each with advantages and disadvantages^33,34^. A self-sizing spiral cuff electrode was chosen (Fig. 10b). Self-sizing spiral cuff electrodes are made of several wraps and do not require any closing system. They have the advantage of snugly fitting around the nerve and adapting their inner diameter to it. The cuff electrode is fabricated according to the method and materials described by Naples et al.^35^. The spiral cuff has the same number of turns (2.5) found to be an ideal balance between a good fitting of the cuff on the nerve and the ability to give way in case of oedema^36^.

**Figure 11:**
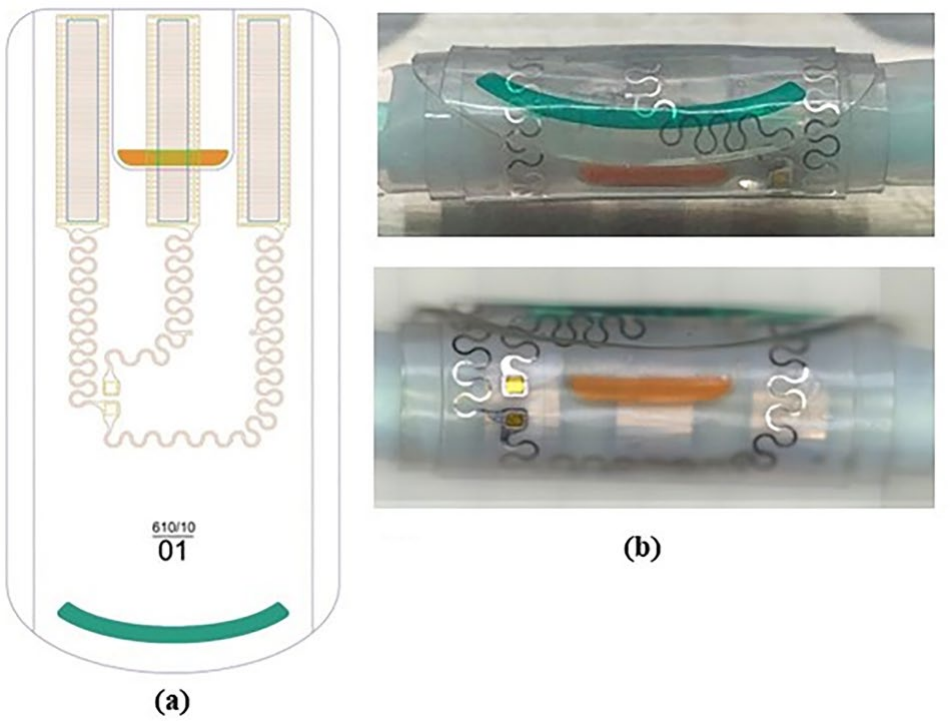
(**a**) Electrode drawing showing electrode contact areas above in a tripolar configuration and conductive paths to the PV die pads. Colored outer (orange) and inner (green) flaps must ease manipulation; (**b**) Electrode photographs.

The cuff electrode consists of several layers of silicone (NuSil™ MED-2000) embedding platinum-iridium contacts and conductive paths (Fig. 11a,b)

Electrode contacts are the electrical metallic surfaces that transmit the current to the nerve. There must be at least two contacts, one anode and one cathode, to allow current to flow. In the tripolar configuration implemented here, a central cathode is located between two interconnected anodes as shown in Fig. 11a. This configuration has the main advantage of confining the current flow within the cuff and thus limiting current spread in the surrounding tissue^37^. Tripolar configuration increases the cuff width (dimension axial to the nerve, Fig. 10a) but, on the other end, it doubles the anode surface, which reduces the load impedance, thus reducing the needed voltage compliance of the PV cells. To ease the placement onto the nerve and the opening and closing of the cuff electrode, outer and inner flaps with color coding are added that can be grabbed with tweezers during surgery.

**Figure 12:**
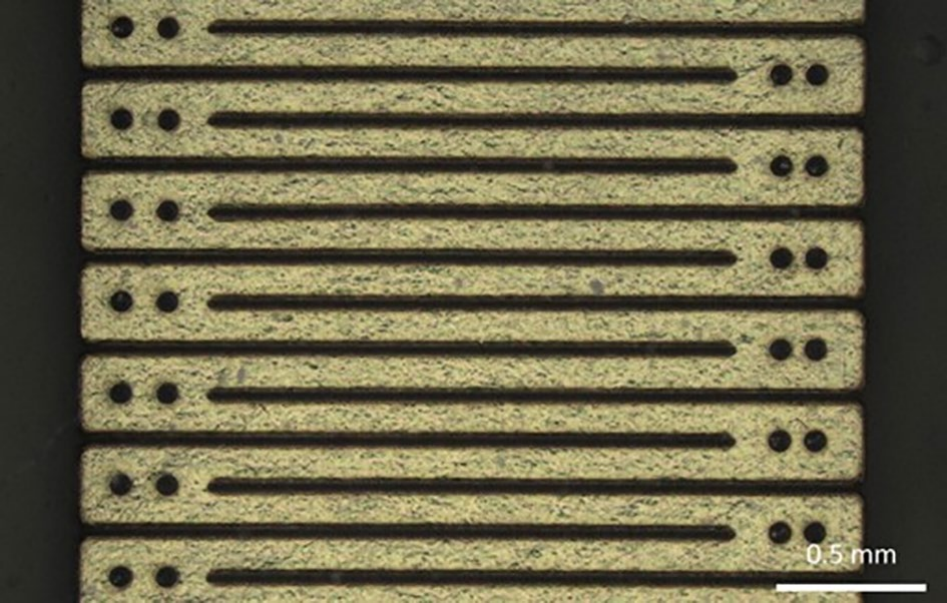
Photograph of one meander-shaped contact of the cuff electrode showing all around the holes made in the contacts to allow anchoring into the silicone sheets.

The contact geometry (Figs. 11a and 12) was carefully designed to meet two constraints. On the one hand, the contact periphery must be maximized as most of the current flows from there^38^. Increasing the contact perimeter while keeping the surface area constant would homogenize the current density and thus decrease the risk of high current density damage.

On the other hand, the electrode stiffness must be minimized to provide a cuff as flexible as possible to reduce nerve damage and to allow the cuff to adapt its shape to the nerve. The meander shape meets these two criteria^39^. Similarly, conductive paths to the die contacts also show curvy features.

### Neurostimulator electronics

**Figure 13:**
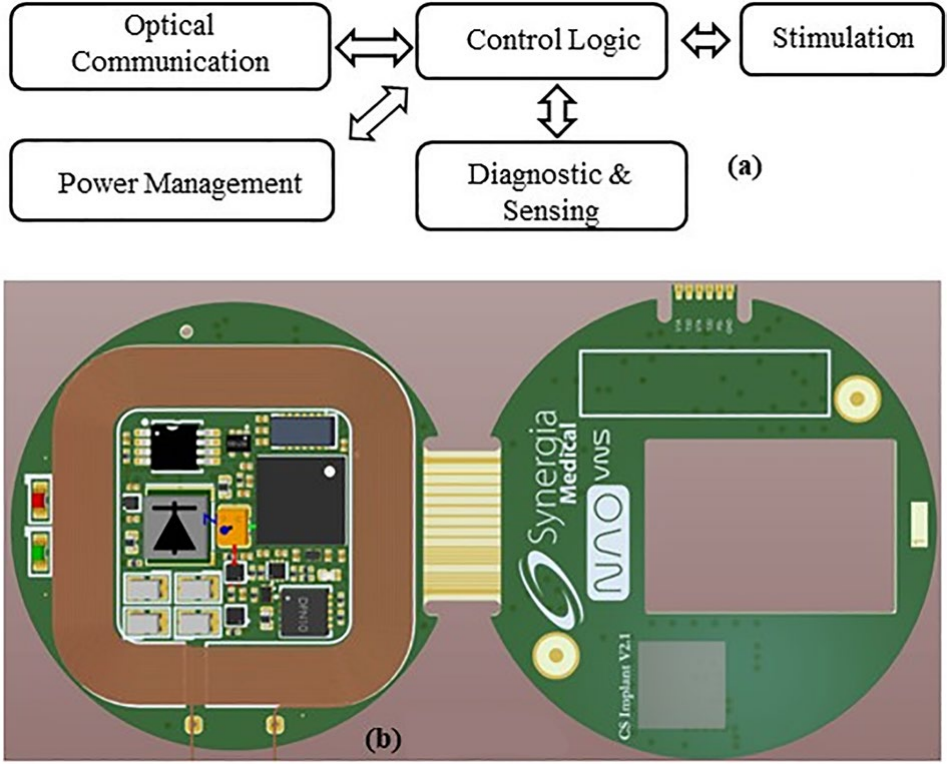
(**a**) IPG main architecture and (**b**) IPG assembled PCB.

The IPG electronics (Fig. 13a) consists of several main elements: a control logic based on a microcontroller, a stimulation circuitry, sensing functions, an optical communication unit (for data exchange with external controller and programmer devices) and a power management unit (including a rechargeable power source, power supplies and an inductive charging circuitry to replenish the implant power source thanks to an external charger).

The IPG electronics are mainly made of commercially available electronic components mounted on FR4 PCB (Fig. 13b) which have been carefully chosen according to a thorough comparison based on their electrical properties, size and power consumption. The different main parts of the implant electronics are listed and detailed hereafter.

#### Power source and inductive charging

A rechargeable battery was chosen to cope with the disadvantages of primary (non-rechargeable) batteries: mainly their bulkiness and the invasiveness and economic impact of their replacement surgeries. The rechargeable battery approach also lifts the hurdle of power limitation when it comes to adding complexity, mathematical power and sensing to a neurostimulator. Choosing the proper battery is a trade-off between small size, large capacity, short charging time and medical qualification. The lifetime of the batteries is also of great importance: the capacity and internal resistance (which should be as small as possible) should not degrade too much or too fast with their number of charging cycles. Also, zero-voltage tolerant batteries would be preferred as they do not require monitoring during shelf-life, and it would reduce the risks of battery failure if not recharged on time. Among the available batteries, none offers a recommended maximum charging rate above C, meaning that at least 1 h is necessary to reach full charge. This limitation led us to look for other alternatives. An off-the-shelf 2.7 V/24 mAh lithium-titanium-oxide (LTO) battery is used with the advantage of being fast to charge (10C, 6 min for a full recharge), with 200 milliOhms equivalent series resistance, high safety and long cycle life above 5000 cycles for 90% of the initial capacity. This rechargeable battery is used for the first prototype and will be replaced in the near future by a custom battery currently under development.

Inductive charging was developed based on a class E driver able to provide at a frequency of 6.78 MHz up to 200 mA of recharging current within a distance range of 0 to 2.5 cm of implantation depth.

#### Control logic

The neurostimulator is a microcontroller-based embedded system capable of delivering the intended therapy, communicating with external parts, sensing physiological parameters and managing a complex power management system with different supply levels and recharging capability. Hence, the control logic unit is a central element in the design. Among the available microcontrollers, several meet the required features of ultra-low power consumption, required number of ports (GPIO) and tiny package. A 16-bit microcontroller has been chosen because it fits these requirements and includes an IrDA module for easy control and interfacing with the optical communication parts, high performance ADC, real-time clock and timers and integrated hardware 128-Bit & 256-Bit AES-128 cryptography.

#### Stimulation circuitry

**Figure 14:**
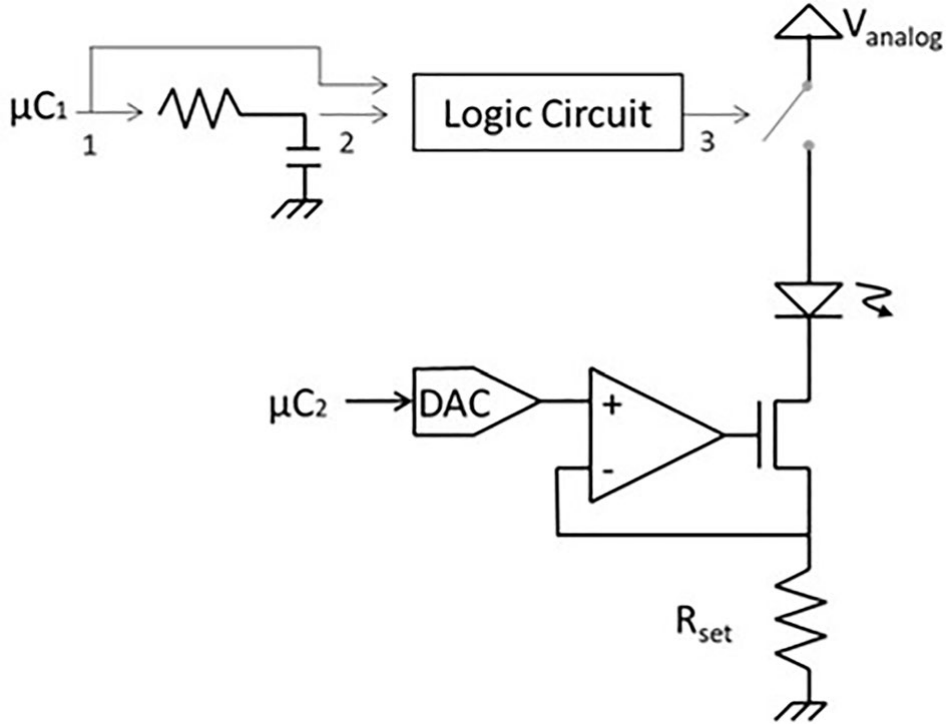
Electrical schematic of the programmable current source driving each VCSEL.

The IPG contains two programmable current sources that respectively drive the VCSELs to generate optical power for the stimulation and recovery currents at the photovoltaic die. Figure 14 shows the equivalent circuit of one of these drivers. The voltage output *V* from a digital-to-analog converter (DAC), is converted by a voltage-to-current converter to drive the optical source with a current *V/R*_*set*_. A switch is present in the current path and acts as a safety feature against mis-programming or hardware failure: based on a resettable delay, it ensures that no prolonged DC current can be generated at the electrode site, engendering electrode corrosion or water electrolysis.

The particularity of this design resides in the opto-isolation provided by the optical means. So, the power supply is the same for each driver, even if they generate opposite currents at the electrode. A 3.5 V power supply is sufficient to drive both VCSELs respectively with up to 100 mA for stimulation and 20 mA for recovery, generating pulses of current with 12-bit resolution, settling time below 10 µs and a minimum timestep of 50 µs.

#### Optical communication

Wireless communication between the implant and the external devices has become essential for physicians and patients to collect data and control implant performances in the frame of personalized therapy. Typically, wireless communication is performed by inductive link modulation or RF transmission. Inductive communication leads to a low data rate while RF transmission is power consuming, requires an external antenna and is prone to hacking.

Among the wireless communication methods that have been investigated to communicate with implants, we chose to develop an optical system based on infrared data transmission at 850 nm, using the IrDA module of the microcontroller and an IrDA transceiver at 115,200 bauds which integrates a signal processing unit at the receiver and a LED driver at the transmitter. The main advantage of optical transmission is the higher data rates, immunity against electromagnetic interference and limited hacking possibilities thanks to the required close contact with the patient. Optical transmission is possible thanks to the optically transparent encapsulation of the IPG. A proprietary communication error-free protocol was developed, including AES-128 cryptography.

#### Sensing functions

Adding sensing functions at the IPG aims to improve the therapy’s effectiveness and the safety by providing essential information on the patient's health and the implant functionality. Apart from the electrode current feedback already described, the sensing functions included in the IPG are a magnetic field detector, for example to sense MRI proximity, and an optical heart rate monitor sensor solution able to sense heart rate and SpO_2_, taking advantage of the transparent encapsulation.

### IPG encapsulation

**Figure 15:**
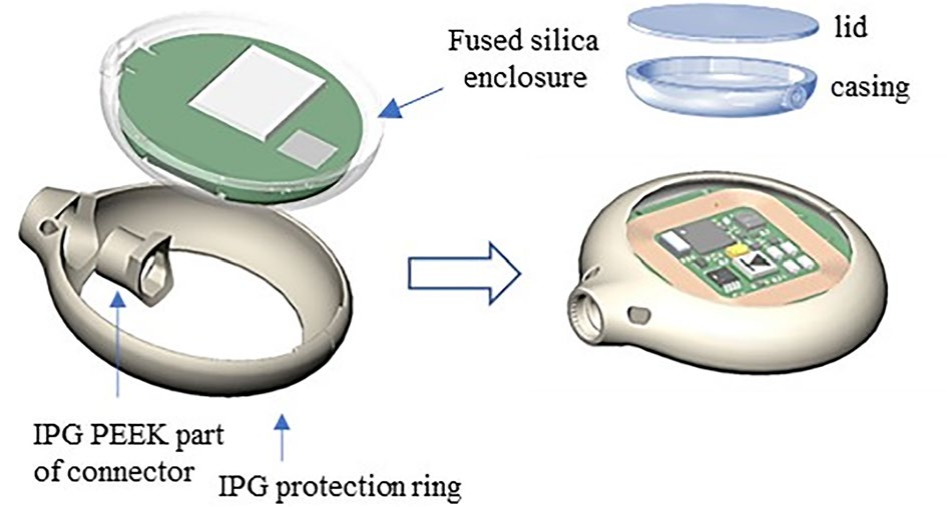
IPG showing electronics potted within a fused silica enclosure, sealed with a lid and further protected with a PEEK ring and a silicone coating.

The encapsulation is defined as the hermetic mechanical protection surrounding the IPG. It consists of several elements (Fig. 15).

The main enclosure is made of transparent fused silica using a unique technology of so-called selective laser induced etching (SLE), using femtosecond lasers^40^. Selective laser-induced etching (SLE) is a two-step process to produce 3D structures in transparent materials: first, a block of transparent fused silica glass is modified internally by laser irradiation and, second, the modified material is selectively removed by wet chemical etching resulting in the development of the 3D product with micrometer accuracy. The electronics of the IPG is placed inside this fused silica enclosure, then reinforced with resin for additional protection and the two fused silica pieces are hermetically sealed with femtosecond laser welding^41,42^. This type of welding has the essential advantage of limited heat generation, which is crucial due to the device's size and the electronics' proximity. The resulting enclosure is further protected with a ring of PEEK biocompatible polymer and a transparent silicone layer.

Transparency of the encapsulation brings the possibility of optical biosensing, optical data and power transmission, optical positioning^43^ and, being nonmetallic, the possibility of integrating the receiver coil together with the electronics.

## Results

The NAO∙VNS System is a Class III implantable device, the most stringent class in terms of medical devices safety and regulatory constraints. The device consists of implantable elements, external accessories for charging, programming and communication devices and surgical ancillaries. It is designed to meet the essential requirements of the European MDR and FDA regulations. Compliance with these requirements (Global Safety and Performance Requirements, GSPR) must be proven by a set of tests that the device must undergo. The higher the device class, the greater the risks associated with its use and the more important and stricter are the associated safety and functionality tests.

Many of these are described in technical and medical standards. The main references are ISO 14708 for the implantable parts, IEC 60601 for the external parts, ISO 10993 for biocompatibility aspects, IEC 62304 for software aspects and ISO/IEC 10974 to establish safety and compatibility with MRI. A description of these is beyond the scope of this paper. Here, we focus on the main results obtained for the optical chain and their positive impact on MRI safety and compatibility.

### Optoelectronic chain for stimulation

#### Photovoltaic cells

**Figure 16:**
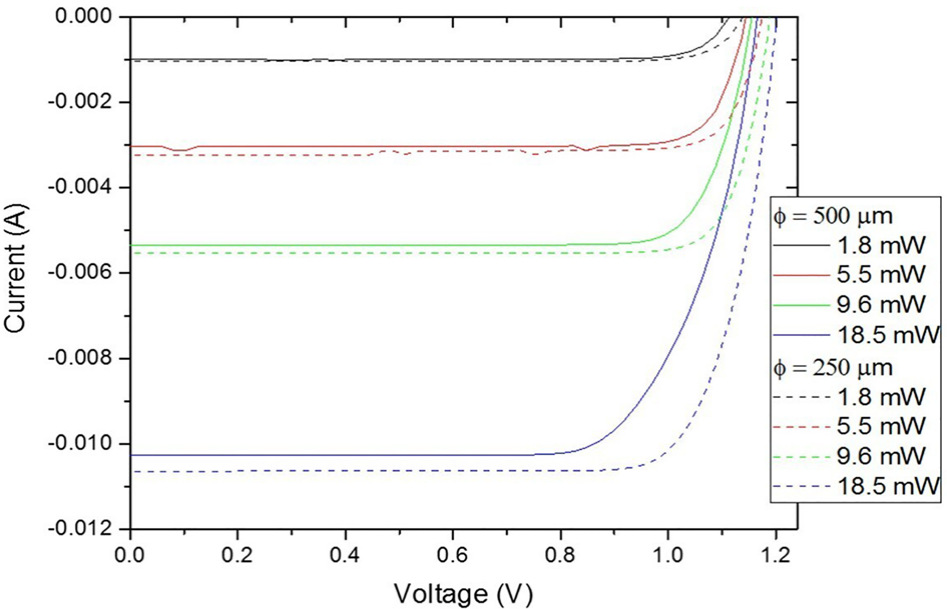
IV curve for 500 and 250 μm diameter single PV cells under 850 nm illumination at different incident optical powers.

The single element PV cell reaches 58% of conversion efficiency, close to the maximum found in the state of the art^44,45^ while multiple segmented cells reach around 54%. Figure 16 shows the current–voltage (IV) curve for 500 and 250 μm diameter single PV cells under 850 nm illumination at different incident optical powers, reaching more than 10 mA of current for 18.5 mW. The flat part of the curve is the ‘current source’ domain, which varies with the programmed optical power.

**Figure 17:**
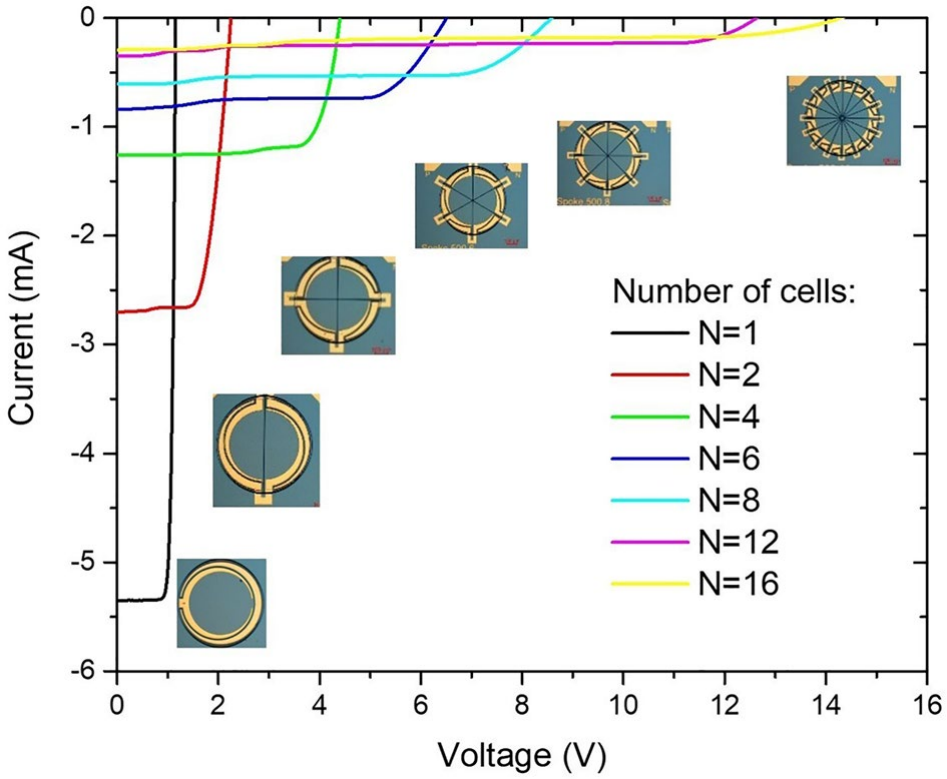
IV curves of different cell configurations where up to 16 PV cells are connected in series.

Cells are connected in series to allow higher voltage compliance of the current source. Figure 17 shows an example of different configurations of up to 16 cells in series. As the number of cells increases, their individual size decreases leading to a predominant effect of the perimeter recombination, explaining the loss of the open circuit voltage Voc for the largest numbers of cells (only 14 V for N = 16 for instance). Besides, an increase of series resistance is observed for the large number, limiting the potential of application for high current.

**Figure 18:**
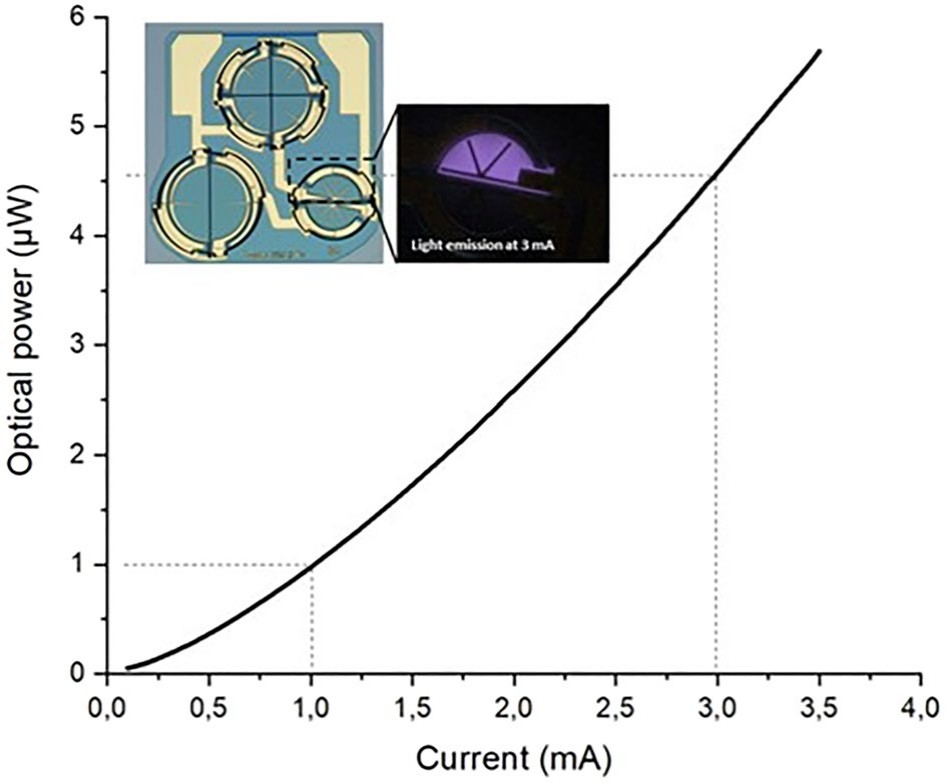
Coupled power – current (LI) curve from the LED with 4.5 µW of optical power generated with 3 mA.

The feedback LEDs were also optimized and measured in terms of performance (Fig. 18). The resulting Wall-Plug Efficiency (WPE) of 0.02% is poor due to limited light extraction and coupling to the fiber, but sufficient for their purpose as a feedback of the current flowing through the current source.

#### Optical fiber

**Figure 19:**
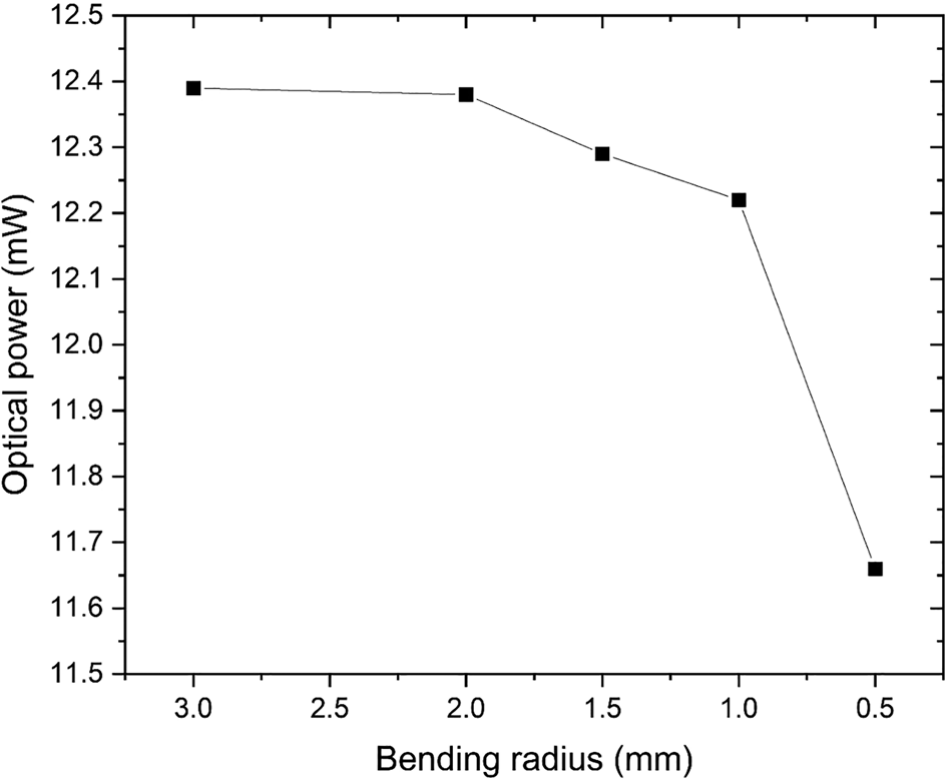
Transmitted optical power variation versus bending radius.

The custom optical fiber was characterized using the cut-back method^21^, showing around 3 dB/m of optical power absorption. Aging tests were performed in a physiological water bath with no noticeable change in the optical properties. The fiber high NA allows very tight bends. Decreasing the bend radius down to 1 mm did result in negligible optical power loss as seen in Fig. 19.

The fiber was repeatedly tested for 82,000 flexion cycles, in compliance with standard ISO14708-1 section 23. The result was a maximum power loss of 3.1% observed on 5 complete optical leads tested, with 3 optical fibers per lead.

#### Overall optical chain efficiency and power requirements

The overall optical chain incurs losses at each stage of the transmission of the optical power from the optical source to the photovoltaic cell. These losses are described by an efficiency ratio (output/input optical power), the overall performance being calculated as the product of each chain component efficiency. A first type of losses is the fused silica interface reflection: the optical beam crosses four of these interfaces on its way from the source to the PV cell, leading to 87% transmitted optical power. The source coupling with the optical connector has an estimated 95% efficiency. The optical fiber transmission losses, which are calculated with a lead length of 40 cm, lead to 70% of optical power transmitted in the fiber. Then, at the distal end of the lead, the PV cell misalignment of the output beam onto the PV die also brings additional losses and a minimum efficiency of 85%. Altogether, it leads to an overall optical chain efficiency of 49.2%.

**Figure 20:**
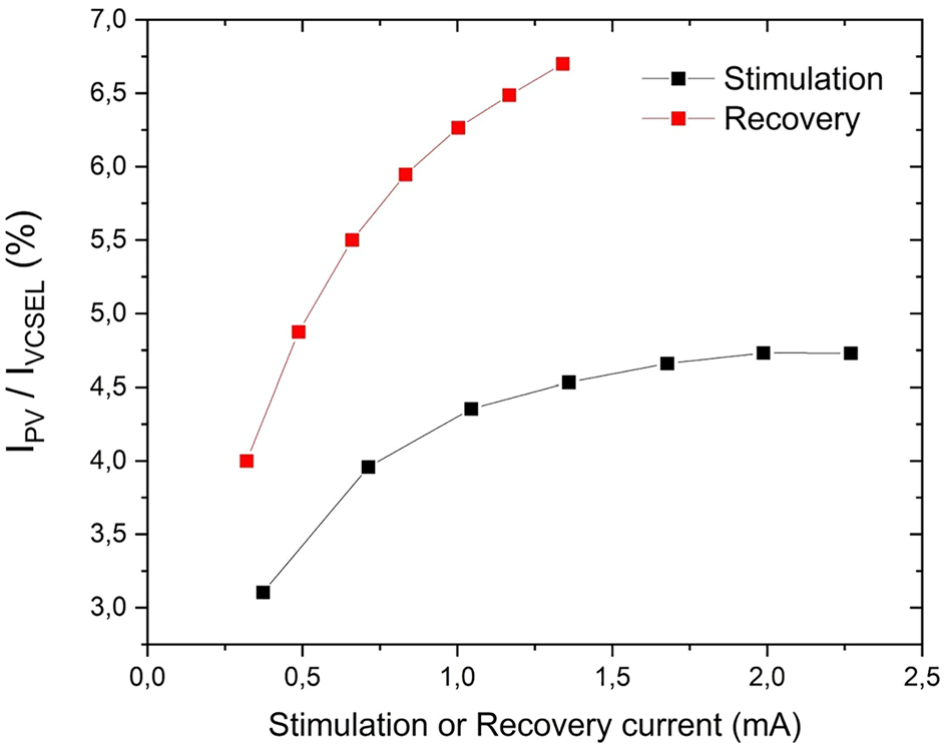
Current ratio between VCSEL driving current and current generated at the electrode for the stimulation and recovery channels; measurements made on a batch of 11 implants, with the graph showing an average value, with a maximum standard deviation of 0.74%.

Considering electrical power requirements, another way to consider the efficiency is the current ratio between the current needed to drive the VCSEL and the one generated at the electrode contacts (Fig. 20) including the optical losses described above as well as VCSEL WPE and PV cell conversion efficiency. Within the usual range of programmed currents, it provides a current ratio between 3 to 7%, which is lower to traditional electrically transmitted stimulation but perfectly acceptable for a rechargeable system. As an example of VNS standard parameters, 750 µA, 250 µs and 30 Hz with time ON of 30 s and time OFF of 300 s^46^, the calculated autonomy between IPG recharge sessions is 7 days.

### MRI safety and compatibility

When introduced into the MRI environment, a device can undergo a malfunction, intense heating, or even be subjected to large forces. These interactions depend on the material, the volume, the shape, the position, the orientation and the distance between the device and the image area. The ISO/TS10974 standard, published in 2018, describes the potential interactions corresponding to various MRI output fields. The MRI machine has a powerful magnetic field *B0,* called static field, produced by a superconducting magnet, generating a strong, uniform magnetic field that polarizes the hydrogen nuclei in the body's tissues. Gradient coils create weaker time-varying magnetic gradient fields *G* which are used to spatially encode the signal from the body. Radiofrequency coils *RF* are used to transmit pulses into the body where they will excite the hydrogen nuclei, which will, in turn, produce measurable signals, collected through an RF antenna.

ISO/TS10974 provides different testing methods to assess the risks related to these fields and ISO 14708-3 provides acceptance criteria which are that (1) RF and Gradient induced heating of adjacent tissue(s) shall not cause an unacceptable risk (this heating value shall be ideally below 2 °C), (2) magnetically induced force shall be less than the weight of the device, (3) magnetically induced torque shall be less than the worst case gravity induced torque and (4) device malfunction shall not cause an unacceptable risk. The outcome of each test shall not result in an unacceptable risk to the patient. These tests were dispatched between different testing labs, depending on their capabilities, accreditation and facilities.

Test results presented here were partly presented at the International Neuromodulation Conference 2022^47^. They were performed for 1.5 and 3 T MRI scanners, the most critical ones being the potential induced heating due to the presence of the implant.

**Figure 21:**
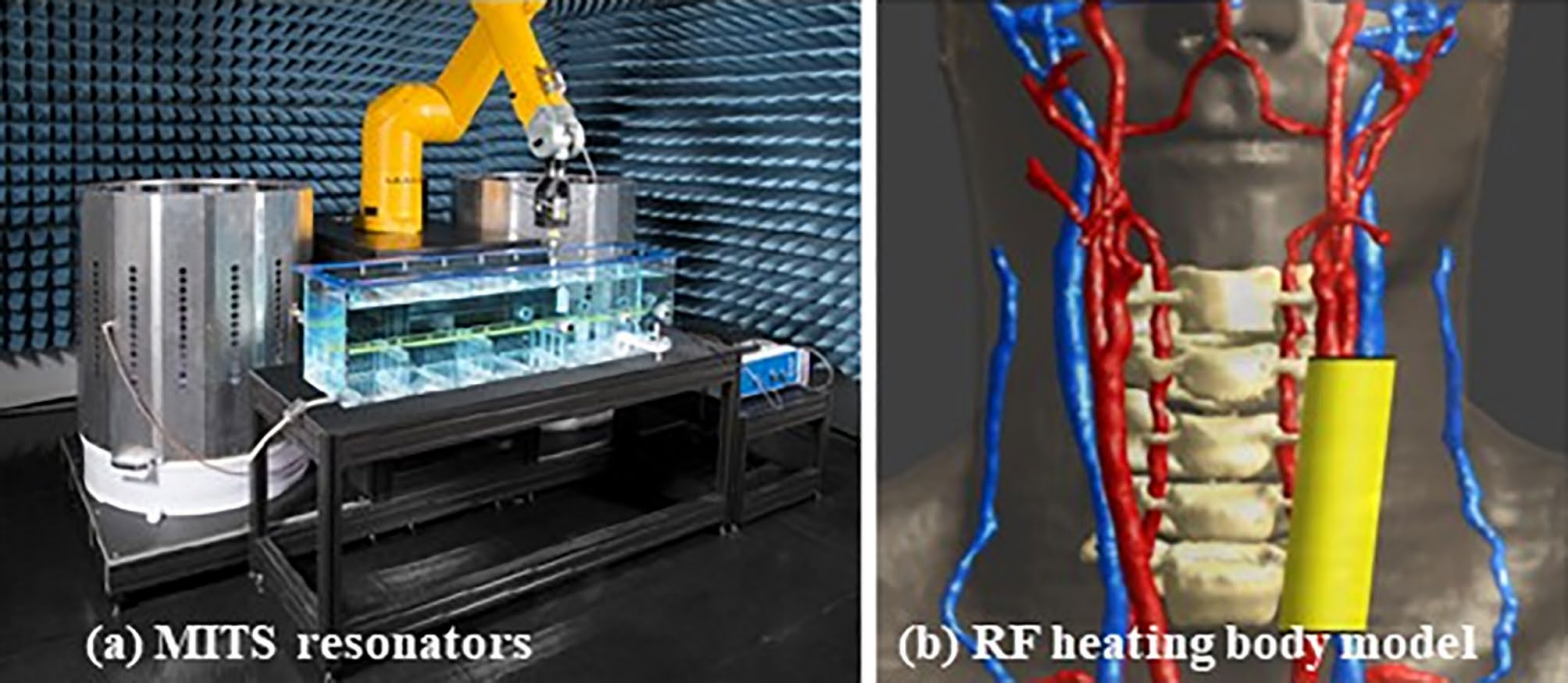
(**a**) Medical Implant Test System MITS involving a resonator, tissue equivalent phantom and robotic arm for automated measurement; (**b**) Body model for simulation showing in yellow the anatomical area used for heating simulation at the electrode (right).

RF-induced heating test strategy is a two-step process. First, numerical models of the electrode and IPG were developed and experimentally validated in two orientations, by radiation tests in high-permittivity tissue simulating medium in the MITS1.5 and MITS3.0 resonators, specifically designed for testing compliance (Fig. 21a) (ZMT, Zurich, Switzerland). Both IPG and electrode are immersed in the medium, subjected to radiation while a motorized calibrated specific absorption rate EX3D probe (ZMT, Zurich, Switzerland) scans the area with the probe tip, 2 mm away of the device surface. The obtained numerical models were validated by comparing the experimental Medical Implant Test System (MITS) measurements with simulations closely mimicking the experimental setup.

**Figure 22:**
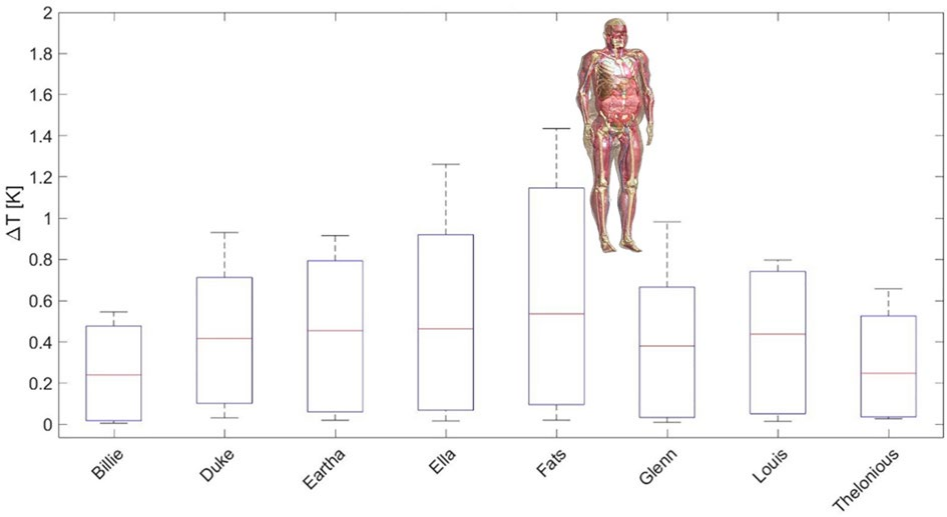
Worst-case temperature rise occurs for the nerve at 64 MHz for the virtual body model Fats.

Second, the validated numerical models were used in Sim4Life (IT’IS, Zurich, Switzerland) to perform numerical full-wave electromagnetic and thermal simulations of maximum MRI exposure conditions in the anatomical regions of interest (Fig. 21b). Cylindrical body coils of different sizes (60, 65 and 70 cm) in quadrature mode were considered for the simulations in combination with a virtual population (IT’IS, Zurich, Switzerland) of different human models. Simulations were performed to determine a conservative upper bound of the maximum temperature increase in the vicinity of the implant caused by the radiofrequency for the relevant scan positions (head and chest scans) for each body coil, polarization and anatomical model at each frequency. As a result, the worst-case temperature rise of the implant in all conditions occurs at the electrode site and for 1.5 T scanners, using the body model Fats (Fig. 22). That worst-case remains below the safety level of 2 °C which means it is safe without any additional study or justification needed.

**Figure 23:**
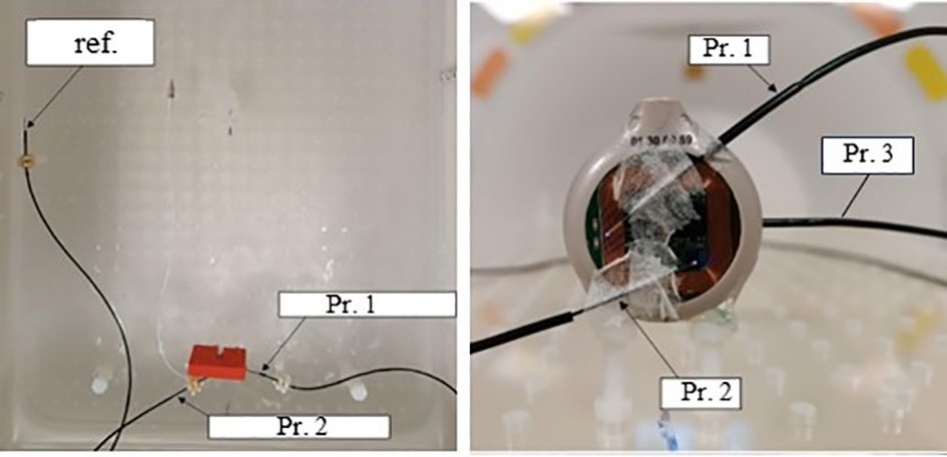
Gradient-induced heating temperature test probes positioned on the (left) IPG surface and (right) cuff electrode central and external contacts.

Gradient-induced heating was conducted following clause 9 Tier 2 of ISO 10974, using a 3 T MR scanner (Siemens, MAGNETOM Prisma 3 T). The implant was placed inside a body phantom 2 cm away from the phantom surface and walls to minimize heat transfer that could impede temperature measurements. Temperature probes have been placed in contact with the IPG surface and electrode contacts (Fig. 23). For the IPG, a preliminary temperature survey has been conducted to find the temperature hot spots at its surface where 3 probes, 2 on the top and 1 at the rear, are placed. The MRI sequence used for the test is an echo planar imaging sequence leading to a clinically relevant test waveform, with radiofrequency field off. Results of the test give a temperature rise for the reference probe of 0.09 ± 1.46 °C, 0.17 ± 1.48 °C for the electrode and 0.28 ± 1.46 °C for the IPG. The maximum temperature increase was then observed for the IPG for an averaged measured exposure value (dB/dt rms) of 32.65 T/s. Considering a maximal exposure limit value of 42.0 T/s according to ISO/TS 10974:2018, it leads to a maximum temperature increase of 0.46 °C, thus also below 2 °C.

Regarding the potential interactions of the magnetic field *B0*, tests were performed with a dedicated set-up in clinical MRI scanners to measure force or torque applied to the implant and they show very weak interactions with no risk for the patient.

Regarding the device malfunction due to the interactions, the implant was placed in a test setup and submitted to MRI scanners combining several scan sequences which are representative of the clinical practice. The total scan exposure for the tested device lasted more than one hour and a half. The implant functionality was tested before, during and after exposure. During the tests, a continuous monitoring of the stimulation delivered by the implant was performed allowing the detection of any potential transient effects or malfunction on the device’s operation. The functionality testing procedure was: (1) IPG functional testing including both communication and recharge features, (2) IPG data integrity testing (3) stimulation output monitoring (stimulation output shall not drift outside tolerances compared to waveform before exposure). No discrepancies were noted compared to the pre-exposure measurements. Specifically, communication being optical, it cannot be disturbed by the MRI fields. Recharging is not possible during the MRI sessions as the external charger is not allowed but tests proved the recharging circuitry poses no threat as the bandwidth used (6.78 MHz) is far away from the 64 and 128 MHz used by MRI scanners.

MRI compatibility was also assessed to characterize the image artefacts generated due to the presence of the implant, following standard ASTM F2119-07 (2013) at 1.5 T and 3 T. Pairs of sagittal acquisition plane imaging were acquired for each frequency encoding direction (anterior to posterior and superior to inferior) and MRI sequence type (spin echo and gradient echo). A first acquisition was performed with the device placed in the field of view, and the second without it. Differences between these two image sets allowed for the characterization of the altered region. Maximum artifact was found in the sagittal plane with spin echo. The IPG induced the largest artifact of dimension 129 mm length on 46 mm width while the lead did not induce any artefact and the one induced by the electrode was strictly confined to the electrode itself.

As a main result, at 1.5 T and 3 T, there is no scan limitation, no limitation for the transmitting coil, no limitation of the operating mode, and no exclusion zone of the body. The neurostimulator will still be labelled as MRI conditional as any AIMD but with no specific conditions to apply for the radiologist.

## Discussion

The designed neurostimulator has been assessed in compliance with ISO/TS10974 for 1.5 T and 3 T MRI scanners. Being an active implantable medical device, it must be labeled either as MRI unsafe, meaning that it poses unacceptable risk to the patient or medical staff, or MRI conditional where it is safe to use with specific conditions to fulfill. While the chosen design minimizes the amount of electrically conductive materials and materials with magnetic properties, electrode contacts are paramagnetic and conductive, and the IPG electronics exhibits conductive tracks and various components with magnetic and conducive properties. Assessments take this into account and prove that the device poses no threat and does not need to answer specific conditions while performing an MRI session. Especially, the RF heating impact remains very low with an increase of temperature around the implant below 2 °C whatever the MRI settings used. It is to be noted that ISO/TS10974 primarily focuses on testing with body coils commonly used in MRI scans, such as those for whole-body imaging or specific body regions, and it does not address testing with local coils such as head coils. If part of the implant is enclosed within the coil scanning area, additional testing and/or calculation might be required to use these coils as results may not directly translate to the use of local head coils due to differences in coil design, field strength and imaging parameters.

In comparison with a classical neurostimulator composed of an IPG with a titanium casing and a lead incorporating wires, the NAO implantable neurostimulation platform brings a tremendous improvement in safety when it comes to undergoing an MRI. Scanning of the full body region is allowed without limitation for the transmitting coil while a classical scheme applies body region restrictions. There is also no scan operating mode limitation nor time limitation, allowing to improve image resolution and quality. In case of a detected broken lead, MRI is forbidden for a classical scheme while it has no impact with an optical lead. Finally, the device can function properly during the MRI session, removing the need to shut down and restart the device before and after the session.

The neurostimulator has been validated functionally and with lab test benches. Biocompatibility studies were performed, and the implant was tested through preclinical studies with sheep.

## Conclusion

The NAO∙VNS is the first-of-its-kind implantable neurostimulator using an optoelectronic approach. It includes an IPG, and optical lead composed of optical fibers. The IPG generates optical pulses, and its components are protected by a hermetically sealed encapsulation made of glass. Optical pulses conveying from the IPG to the electrode are converted back into electrical stimuli by ultra-miniature photovoltaic cells. This approach allows implanted patients to safely undergo MRI (1.5 T and 3 T) without specific or additional precaution.

The NAO platform has been designed to fulfill the requirements of a class III long-term implantable medical device, following the European Medical Device Regulation (MDR) and FDA regulations. The platform was first adapted for the treatment of epilepsy through vagus nerve stimulation and developed for long-term human implantation. Synergia Medical envisages a first human clinical trial for the NAO∙VNS neurostimulator in the coming months.

Future development of the NAO∙VNS neurostimulator involves the use of optical sensing such as heartbeat and SpO_2_ detection paving the way to close loop therapy. As the device can safely function under MRI, it also offers the possibility of functional magnetic resonance imaging (fMRI). This technique is based on the principle that when a particular region of the brain becomes active, there is an increased demand for oxygenated blood in that area. Imaging the blood flow thus provides a representation of brain activity localization that can be used in several ways. For example, with fMRI, epileptologists could identify in real time the specific brain regions that are modulated by vagus nerve stimulation. This can help understanding the neural circuitry involved but also evaluate optimal for individual patients’ stimulation parameters (e.g., intensity, frequency). It can also be used to monitor therapeutic progress. For the purpose of fMRI, additional optically based accessories are currently under development to allow communication between the implanted neurostimulator within the MRI room and the external control room. Assessing the compatibility and safety with 7 T scanners is also envisaged.

Many applications would benefit from a neurostimulator that is safe under MRI without any limitations. For example, Deep Brain Stimulation (DBS) is one of the most suitable applications, as DBS involves using electrodes implanted in specific brain regions to modulate abnormal neural activity. It has been used to treat various neurological and psychiatric conditions, such as epilepsy, Parkinson's disease, essential tremor, and obsessive–compulsive disorder. MRI brain scans are paramount for medical diagnosis and follow-up for these patients, but this is made extremely difficult, dangerous or impossible mainly due to the excessive heating due to the presence of the electrode. Again, an implantable neurostimulator compatible with fMRI would not only allow for brain imaging but also for imaging during stimulation, enabling researchers and practitioners to investigate the immediate effects of DBS on brain activity.
